# Human Small Heat Shock Protein B8 Inhibits Protein Aggregation without Affecting the Native Folding Process

**DOI:** 10.1021/jacs.3c02022

**Published:** 2023-07-06

**Authors:** Dhawal Choudhary, Laura Mediani, Mario J. Avellaneda, Sveinn Bjarnason, Simon Alberti, Edgar E. Boczek, Pétur O. Heidarsson, Alessandro Mossa, Serena Carra, Sander J. Tans, Ciro Cecconi

**Affiliations:** † Department of Physics, Informatics and Mathematics, University of Modena and Reggio Emilia, 41125 Modena, Italy; ‡ Center S3, CNR Institute Nanoscience, Via Campi 213/A, 41125 Modena, Italy; § Department of Biomedical, Metabolic and Neural Sciences, and Centre for Neuroscience and Neurotechnology, University of Modena and Reggio Emilia, Via G. Campi 287, 41125 Modena, Italy; ∥ FOM Institute AMOLF, Science Park 104, 1098 XG Amsterdam, The Netherlands; ⊥ Department of Biochemistry, Science Institute, University of Iceland, Sturlugata 7, 102 Reykjavík, Iceland; # Max Planck Institute of Molecular Cell Biology and Genetics, Pfotenhauerstr. 108, D-01307 Dresden, Germany; ∇ INFN Firenze, Via Sansone 1, 50019 Sesto Fiorentino, Italy

## Abstract

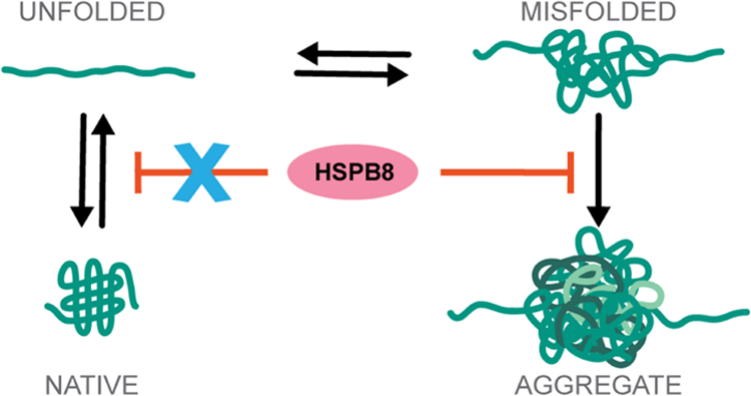

Small Heat Shock Proteins (sHSPs) are key components of our Protein Quality Control system and are thought to act as reservoirs that neutralize irreversible protein aggregation. Yet, sHSPs can also act as sequestrases, promoting protein sequestration into aggregates, thus challenging our understanding of their exact mechanisms of action. Here, we employ optical tweezers to explore the mechanisms of action of the human small heat shock protein HSPB8 and its pathogenic mutant K141E, which is associated with neuromuscular disease. Through single-molecule manipulation experiments, we studied how HSPB8 and its K141E mutant affect the refolding and aggregation processes of the maltose binding protein. Our data show that HSPB8 selectively suppresses protein aggregation without affecting the native folding process. This anti-aggregation mechanism is distinct from previous models that rely on the stabilization of unfolded polypeptide chains or partially folded structures, as has been reported for other chaperones. Rather, it appears that HSPB8 selectively recognizes and binds to aggregated species formed at the early stages of aggregation, preventing them from growing into larger aggregated structures. Consistently, the K141E mutation specifically targets the affinity for aggregated structures without impacting native folding, and hence impairs its anti-aggregation activity.

## Introduction

Molecular chaperones are an evolutionarily conserved family of proteins that form an integral component of Protein Quality Control system, and play a key role in maintaining cellular proteostasis.^1−5^ The functional repertoire of these proteins is diverse and includes actions such as identifying terminally dysfunctional proteins for proteolytic degradation, suppressing the aggregation of misfolded/unfolded proteins and aiding the de novo folding or assembly of other proteins. The disruption of proper protein folding contributes to protein homeostasis imbalance and has been implicated in various neurodegenerative disorders, highlighting the importance of molecular chaperones for life at the cellular and organismal levels.^6−8^

One major group of molecular chaperones are the Heat Shock Proteins (HSP), which are upregulated under stressful conditions that promote protein denaturation and misfolding, and are known to counter protein aggregation in the cellular environment.^1,4,9,10^ HSPs are further subdivided by their molecular weight in the following subfamilies: HSP100, HSP90, HSP70, HSP60, HSP40, and small HSPs, with a molecular weight ranging from ca 15 to 40 kDa.^4,9,10^ Since their discovery more than four decades ago, a vast amount of literature has been dedicated to understanding HSPs’ structural conformations, their mechanisms of action, and the biological processes in which they are involved.^1,2,4,11−14^ HSPs interact with a wide variety of substrates and mediate protein function and activity, thus regulating, directly or indirectly, most aspects of cell biology.

Our understanding of the chaperone activity of HSPs has increased rapidly over the past decades.^11,15−20^ However, it has been challenging to elucidate how substrate conformations are affected, owing to their dynamic and heterogeneous nature. Hence, the core mechanisms of action of many HSPs remain poorly understood. Single-molecule manipulation techniques, such as optical tweezers (OT), magnetic tweezers (MT), and atomic force microscopy (AFM), have more recently allowed addressing this issue with a completely new approach. Single proteins are mechanically tethered at their N- and C-termini in these experiments, which allow one to follow chaperone-mediated conformational changes in real time by detecting the associated nanometer scale contractions and piconewton tensions within the substrate chain. This approach has revealed various mechanisms that remained hidden in ensemble average techniques.^21−25^ In recent times, OT, MT, and AFM have been successfully employed to decipher the structural dynamics and functional mechanisms of HSPs such as HSP100,^26^ HSP90,^27,28^ and HSP70,^29−33^ and of small HSPs, such as yeast HSP42^34^ and archaea HSP16.5.^35−38^

The present study employs OT assays to explore the functional profile of the human small heat shock protein HSPB8. HSPB8, also known as HSP22, is one of the 10 members of the human small HSP (HSPB) family and is expressed widely in striated and smooth muscles, as well as in motoneurons in the spinal cord.^39,40^ Similar to the other members of the HSPB family, HSPB8 contains a conserved and structured α-crystallin domain and flexible N-terminal and C-terminal regions, which are disordered. It can interact with BCL2-associated athanogene 3 (BAG3) and HSP70, forming the HSPB8–BAG3–HSP70 chaperone complex, which favors the autophagy-mediated degradation of a large variety of substrates, including mutated proteins linked to neurodegenerative diseases such as spinal and bulbar muscular atrophy and amyotrophic lateral sclerosis.^41−43^ Studies also show that expression of HSPB8 in motoneurons declines with age specifically leaving them vulnerable to deleterious impacts of protein aggregation.^42^ Additionally, two missense mutations in the α-crystallin domain of HSPB8, namely, HSPB8-K141E and HSPB8-K141N, have been linked to motor neuropathies such as Charcot-Marie-Tooth neuropathy type 2L and other muscular and neuronal disorders.^44,45^ Data obtained in cells, *Drosophila melanogaster*, and test tube support the idea that the K141E and K141N mutations impair HSPB8 chaperone-like and pro-degradation activities.^46−50^ How exactly HSPB8 exerts its chaperone-like and pro-degradation activity is still only in part understood. Although interacting with the ATP-dependent chaperones HSC70/HSPA8 and HSP70'/HSPA1A, which support protein folding, HSPB8 seems to promote protein degradation rather than refolding. HSPB8 pro-degradation activity is thought to be mediated by its interaction with the HSC70/HSP70 co-chaperone BAG3.^51,52^ Concerning the chaperone-like activity, this varies depending on the type of substrate that interacts with HSPB8.^53^ Data obtained using the RNA binding protein fused in sarcoma (FUS) or mutated polyglutamine huntingtin as model proteins, and using wild-type HSPB8 or an HSPB1-HSPB8 chimera, demonstrate that the conserved α-crystallin domain is required for HSPB8 chaperone activity.^50,52^ Yet, whether HSPB8 interacts with similar affinities with unfolded, misfolded, or aggregated substrates and how this influences its chaperone-like activity is unknown.

In this work, we use optical tweezers to study how HSPB8 and its disease-causing mutant HSPB8-K141E affect the aggregation process of the Maltose Binding Protein (MBP). MBP has been often employed as a model system for chaperone-guided folding studies in ensemble-averaged^54−56^ and single-molecule studies,^57,58^ and allows for single-molecule investigation of aggregation when arranged in tandem repeats.^59^ Our results reveal a peculiar chaperone activity of HSPB8: it suppresses aggregation without affecting native folding. Unlike other chaperones tested in ensemble-averaged^60−62^ and single-molecule experiments,^31,57^ HSPB8 does not limit aggregation by stabilizing unfolded polypeptide chains or near-native states of the substrate protein. Rather, it prevents the growth of misfolded conformations into larger structures, likely by interacting with misfolded conformers formed at the onset of aggregation. This interpretation is further supported by the experimental evidence that the K141E mutation directly affects the anti-aggregation activity, but has no effect on the probability of native folding.

## Results

**Figure 1:**
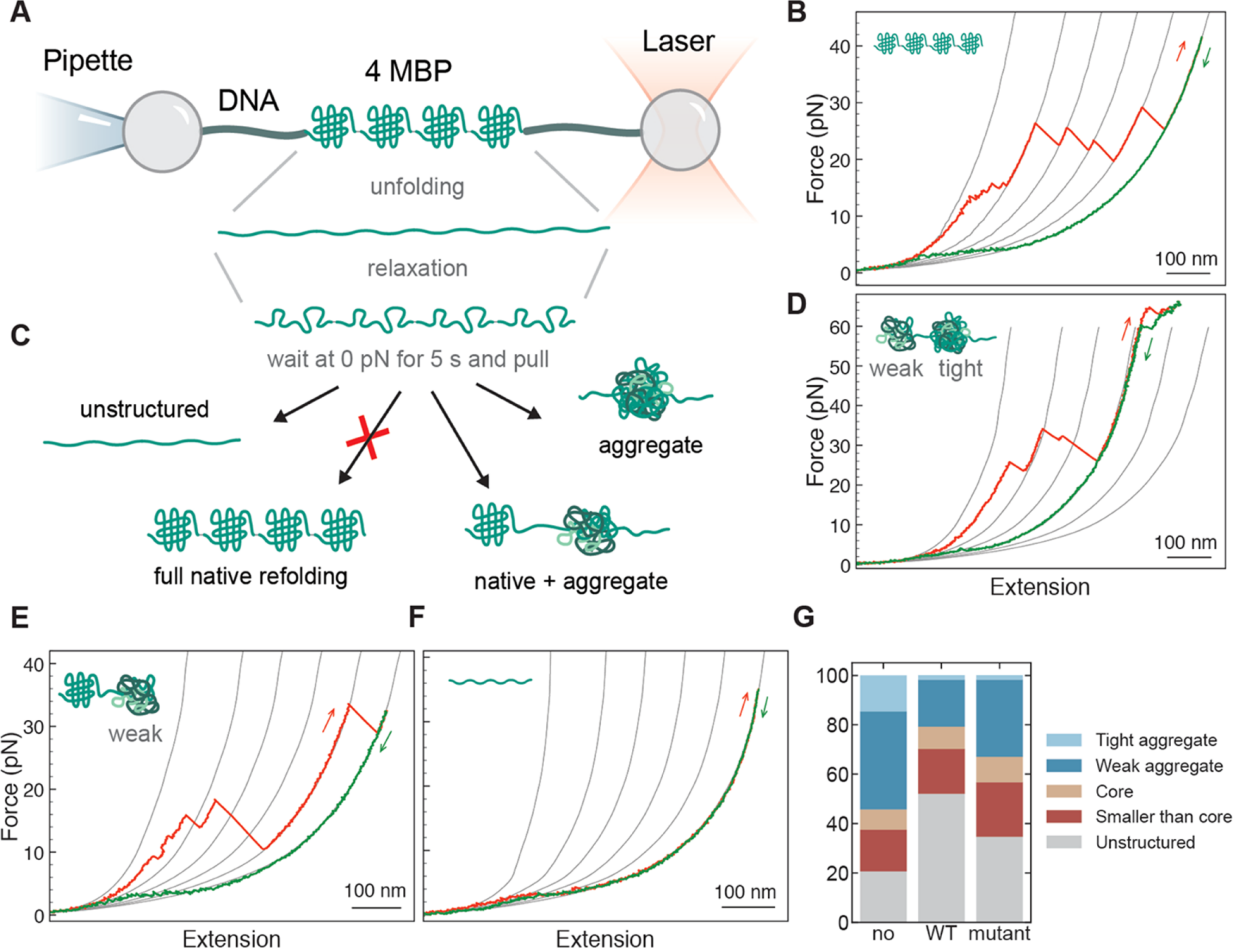
Mechanical manipulation of 4MBP with and without the presence of chaperones. (A) Four maltose binding proteins arranged in tandem (4MBP) are mechanically manipulated with polystyrene beads by means of DNA molecular handles. One bead is held at the end of a micropipette by suction, while the other is held in an optical trap. By moving the beads relative to each other, the protein can be stretched and relaxed, while the molecular extension and the applied force can be measured as described in refs (21, 64). (B) When stretched for the first time, 4MBP starts losing its structure when external α helices unzip from each monomer and unfold. These structural changes generate a 4MBP lengthening of 100 nm that gives rise to a gradual discontinuity in the stretching trace at ∼10 pN (B). At higher forces (∼25 pN), the remaining core structures unfold sequentially giving rise to a sawtooth-like pattern where each rip corresponds to the unfolding of 250–290 aa, as estimated according to the procedure described in the Data Analysis section, where it is also explained the origin of the reference gray lines. (C) After complete denaturation of the 4MBP molecule, the applied force is relaxed and held at 0 pN for 5 s before the molecule is pulled again. During this relaxation period, amino acids from adjacent domains can interact and end up in different molecular states, as depicted in (C). An analysis of the unfolding jumps observed in the second or subsequent stretching traces allowed us to distinguish 5 molecular states: (i) “tight aggregates”, i.e., compact structures that survive at forces larger than 63 pN (D), (ii) “weak aggregates”, related to jumps that involve more than 290 aa ((D) and (E)), (iii) “core-like structures”, related to jumps that involve between 250 and 290 aa (E), (iv) “small structures”, related to jumps that involve less than 250 aa ((D) and (E)), and (v) “unstructured”, all of the amino acids that do not end up into any of the previous categories (F). (G) Percentage of aa that end up in each of these molecular states in the presence of no chaperone (107 traces; 3 individual molecules), HSPB8 (5 μM) (132 traces; 4 molecules), or HSPB8-K141E (5 μM) (190 traces; 11 molecules).

The chaperone activities of HSPB8 and HSPB8-K141E were studied with optical tweezers using four maltose binding proteins arranged in tandem (4MBP) as substrate. Individual 4MBP molecules were tethered to polystyrene beads by means of molecular handles^63^ and then stretched and relaxed multiple times in the absence or presence of HSPB8 or HSPB8-K141E (Figure 1A).

When stretched for the first time, 4MBP starts losing its structure at ∼10 pN as C- and N-terminal helical segments of each monomer detach and unfold. Then, the remaining monomer core structures unfold sequentially at higher forces (∼25 pN) giving rise to a sawtooth pattern characterized by extension increases corresponding each to the unfolding of 250–290 aa (Figure 1B). This sequence of unfolding events takes place only during the first pull. Indeed, when force is relaxed to 0 pN for 5 s^57^ to allow 4MBP refolding, interactions between adjacent domains compete with native folding and the majority of residues end up in non-native conformations that, upon pulling, unfold in a wide range of forces and molecular extensions (Figure 1C). In the absence of chaperone, about ∼50% of the residues aggregate into structures comprising more than one MBP core (more than 290 residues) that upon stretching either unfold before the beginning of DNA overstretching at 63 pN (“weak aggregates”) or do not unfold even at 63 pN (“tight aggregates”) (Figure 1D,E). About 20% of residues avoid aggregation and fold into native core structures (Figure 1E). The rest of the residues either fold into small conformations involving less than 250 residues or remain completely unstructured (Figure 1F,G).

In the presence of wild-type HSPB8, the aggregation propensity of 4MBP strongly decreases. While the first stretching trace remains the same, which indicates that HSPB8 does not interact with natively folded MBP, in subsequent stretching traces after unfolding and relaxation, tight aggregates are hardly observed (less than 2%), and on average, only ∼19% of the residues are forming weak aggregates (Figure 1G). Conversely, the fraction of residues that are either unstructured or form structures smaller than a native core increases from 38% to 70%. Notably however, the frequency of native folding within a 4MBP molecule remains unaffected: 8% without chaperone, 9% with wild-type HSPB8 (Figure 1G).

These observations are puzzling: if aggregation is suppressed because the presence of the chaperone hinders interactions between different segments of the tethered chains, why is native folding not affected? Models previously investigated in relation with other HSPs suggest that these chaperones interact with unfolded peptides.^31,57^ Aggregation suppression and folding are then bound by a trade-off, which is not the case here (Figure 1G). This observation is further supported by the data on HSPB8-K141E, which is less effective in suppressing weak aggregates (31% versus 19% for wild-type HSPB8; 40% in the absence of chaperone), while the frequency of forming native core structures is again not significantly^a^ altered (10% versus 9% for wild-type HSPB8; 8% in the absence of chaperone). We note that the ability to suppress tight aggregates is similar to wild type, while the frequency of unstructured amino acids shows an intermediate value between the cases with and without wild-type HSPB8 (Figure 1G).

Altogether these data show that both HSPB8 and HSPB8-K141E can suppress protein aggregation without affecting native folding. Several molecular mechanisms could explain these highly selective chaperone activities.

**Figure 2:**
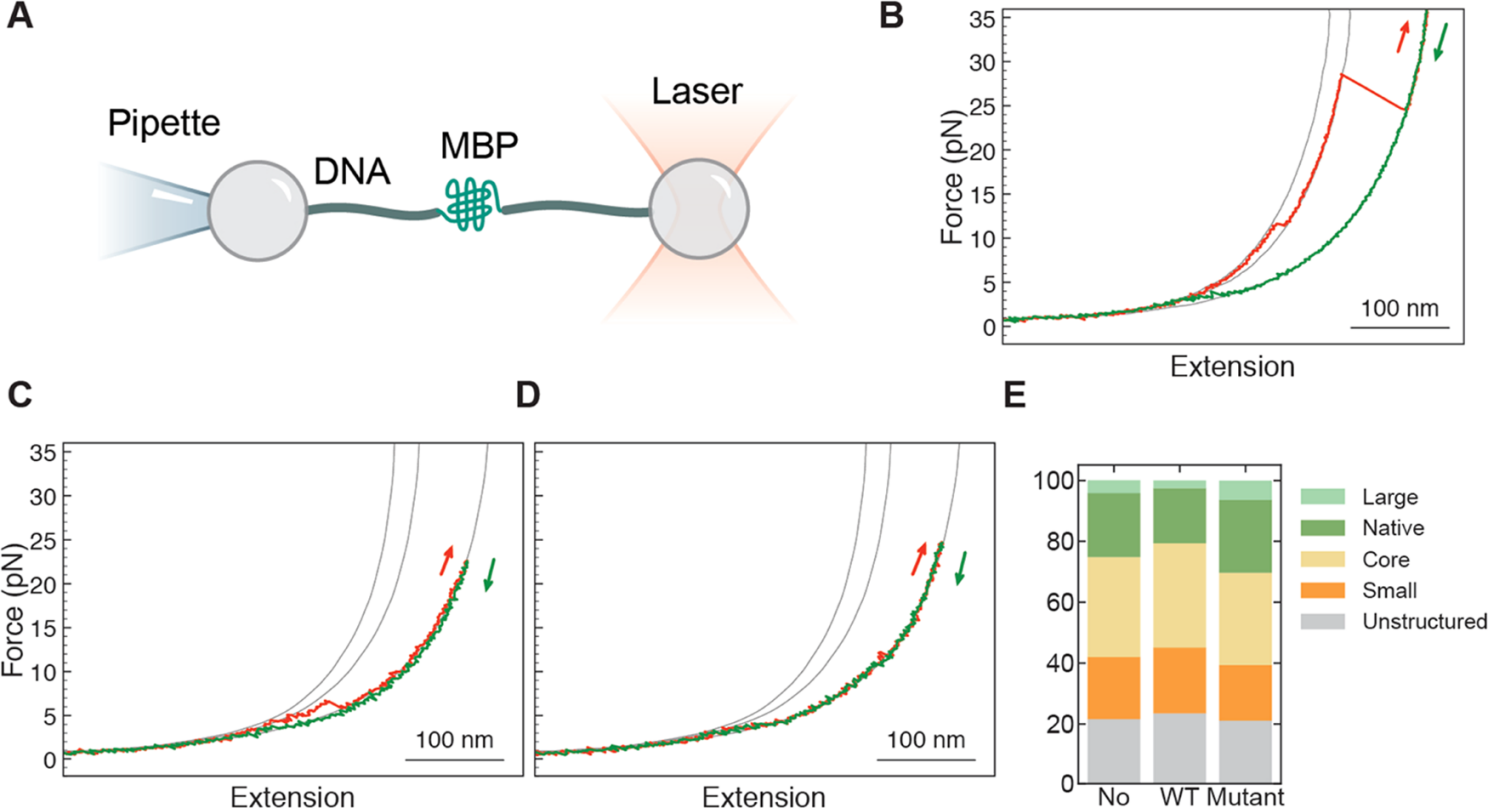
Mechanical manipulation of sMBP with and without the presence of chaperones. (A) A single sMBP is mechanically manipulated within the same experimental setting employed in 4MBP case (Figure 1A). (B–D) Force vs extension curves of stretching-relaxation cycles performed on an sMBP. The gray lines are the reference curves for the elastic behavior of the handles plus a chain of 0, 91, and 370 unfolded amino acids, from left to right respectively, as explained in the Data Analysis section. While the relaxation curves (green) hardly vary, the stretching (red) curves reveal details about the state of the protein. (B) Unfolding of a native state, characterized by the denaturation of α helices around 10 pN, followed by the unfolding of the core structure around 25 pN. (C) Denaturation of a structure clearly smaller than a typical core. (D) Stretching of an unstructured amino acid chain, without any detectable discrete unfolding events. (E) Each stretching trace can be classified into one of 5 categories (defined in the text), using 179 traces and 8 individual molecules for the dataset without chaperone, 214 traces and 6 molecules for the dataset with wild-type HSPB8, and 155 traces and 5 molecules for the dataset with the mutant chaperone. The observed relative proportion is largely unaffected by the presence of HSPB8, both wild type (5 μM) and mutant (5 μM). Note that this chart is based on a classification of traces, unlike Figure 1G which is based on a classification of jump events.

To gain further insight into the HSPB8 mechanism of action, we studied the effect of HSPB8 and HSPB8-K141E on the folding process of single MBP monomers (sMBP). Single sMBP were manipulated as depicted in Figure 2A.

They were stretched and relaxed multiple times with and without the presence of chaperones. In accord with what was observed with 4MBP, the first stretching trace is typically characterized by a small discontinuity at about 10–15 pN, corresponding to the unfolding of external α helices of sMBP, followed by a larger transition around 25 pN, due to the denaturation of the remaining core structure (Figure 2B). Upon relaxation of the force to 0 pN for 5 seconds several possible scenarios are observed during the subsequent stretching process: the unfolding of a core structure, either complemented by external α-helical segments (the trace is then indistinguishable from the first one, and is classified as ″native″) or not (classified as ″core″); the unfolding of structures that are smaller or more fragile than a core (classified as ″small″, Figure 2C), or stretching traces devoid of discernible structures that are unfolded (classified as ″unstructured″, Figure 2D). Albeit rare, we also find traces showing the unfolding of structures larger than a core at low forces (classified as ″large″; data not shown), which could indicate the simultaneous unfolding of the core and external α-helical segments.

**Figure 3:**
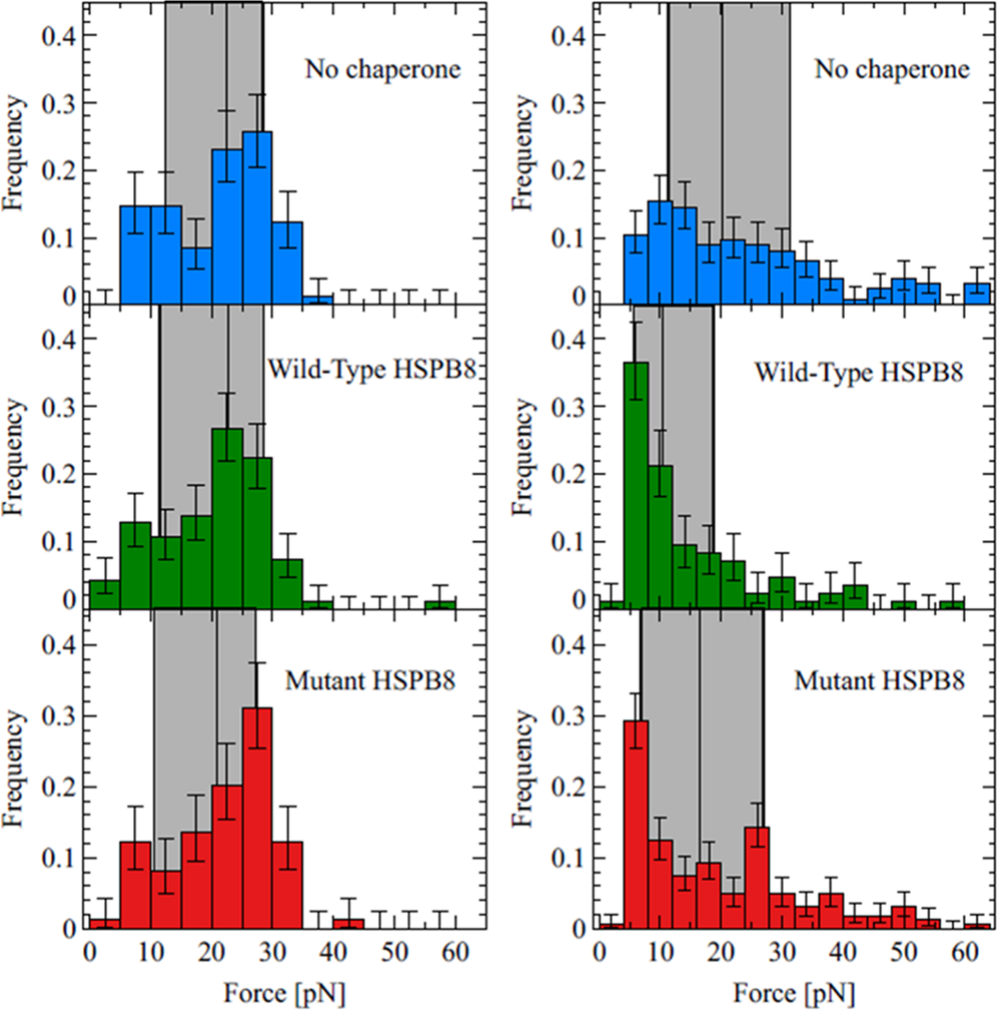
Distribution of unfolding forces of core structures (left column) and weak aggregates (right column), in three distinct experimental settings: 4MBP in the absence of chaperone (top row), in the presence of the wild-type HSPB8 (middle row), or in the presence of the K141E mutant HSPB8 (bottom row). As a guide to the eye, the region between the first and third quartile of the distribution has been highlighted by a gray rectangle. Also, the position of the median is represented by a vertical line. The presence of the chaperone does not affect the mechanical stability of the core structure, while it significantly lowers the breakage force of the weak aggregates, an effect that in the case of the mutant is less pronounced. Histograms are based on 62, 47, and 97 core structures (left column, top to bottom) and on 124, 85, and 161 weak aggregates (right column, top to bottom).

Notably, quantitative analysis of the above categories shows that the presence of HSPB8 WT and K141E does not affect the probability of folding a core (Figure 2E, Table 1, Section 1 in the Supporting Information for details about the statistical significance of data). Nor do the chaperones affect the mechanical stability of (re)folded core structures, as revealed by the unfolding force mean values (Table 2) or distributions (Figure 3, left column). The interaction between HSPB8 and sMBP was also studied by single-molecule FRET experiments. The end-to-end distance and the diffusion properties of fluorescently labeled HSPB8 were probed in the presence and absence of sMBP, confirming that the two proteins do not interact (Figure S1).

**Table 1:** Monomer Data: Classification of Traces^a^

kind	*No HSPB8*	*HSPB8-WT*	*HSPB8-mut*
large	4.2% (+2.2–1.5%)	2.5% (+1.6–1.1%)	6.3% (+2.8–2.0%)
native	21.0% (+3.6–3.3%)	18.1% (+3.1–2.8%)	23.9% (+4.1–3.7%)
core	32.9% (+4.0–3.8%)	34.3% (+3.6–3.5%)	30.3% (+4.3–4.0%)
small	20.4% (+3.6–3.2%)	21.6% (+3.3–3.0%)	18.3% (+3.8–3.3%)
unstructured	21.6% (+3.7–3.3%)	23.5% (+3.3–3.1%)	21.1% (+4.0–3.5%)

a The errors signal the 68.3% central confidence interval.

**Table 2:** Unfolding Force of Core Structures

	monomer	tetramer
*No HSPB8*	(21.8 ± 0.9)pN	(21.4 ± 1.5)pN
*HSPB8-WT*	(21.6 ± 0.9)pN	(20.7 ± 1.3)pN
*HSPB8-mut*	(21.8 ± 0.9)pN	(19.6 ± 1.0)pN

These results are in sharp contrast to the behavior observed in similar experiments on other heat shock proteins,^34,58^ and significantly reduce the range of plausible hypotheses about the microscopic details of the chaperone–substrate interaction for this system. At the same time, these findings fully support our presented observations on 4MBP, which similarly showed HSPB8 avoided interference with MBP core refolding (Figure 1G).

How can HSPB8 prevent aggregation without affecting the formation of the core of MBP in a detectable way? The explanation that best matches our observations is that HSPB8 interacts with off-pathway (misfolded) structures formed at the onset of aggregation, and hence prevents their growth into larger formations, while not (or very weakly) interacting with structures that are on-pathway for the formation of the native core. This hypothesis is indeed consistent with the finding that in the presence of HSPB8 the weak aggregates are smaller in size (Figure S2) and are disrupted at lower forces (Table 3 and Figure 3, right column), even though HSPB8 does not interact detectably with natively folded cores (Figures 2E, S3, and S4). It is interesting to note that an instance of HSPB8 preventing the growth of aggregates has also been observed in experiments with α-synuclein.^65^

**Table 3:** Size, Breakage Force, and Frequency of Weak Aggregates

	size	breakage force	no. of aggregates per trace
*No HSPB8*	(486 ± 16)aa	(23.4 ± 1.3)pN	1.16
*HSPB8-WT*	(417 ± 13)aa	(14.5 ± 1.3)pN	0.64
*HSPB8-mut*	(490 ± 18)aa	(19.1 ± 1.1)pN	0.85

## Discussion

Deciphering the molecular mechanisms mediating the interaction between chaperones and their client proteins is critical to understanding how cellular proteostasis is maintained. Here we show how the use of a single-molecule technique can give us unique insight into the microscopic details of the protein aggregation suppression activity accomplished by HSPB8.

In the most common model of aggregation suppression,^66−68^ and also proposed for the *Escherichia coli* chaperone SecB,^57^ chaperones bind and stabilize the unfolded state of the substrate protein, thus hindering structure formation in general. The data presented for HSPB8 rather point to a different mechanism, as the probabilities for sMBP and 4MBP to fold into their core structure are not affected significantly (Figures 1G and 2E), and indeed the probability for sMBP to remain unstructured also remained unchanged. According to our data, interactions between HSPB8 and the denatured amino acid chain of sMBP during force relaxation do not detectably alter the native folding mechanism of the protein, nor stabilize its unfolded state.

Alternatively, HSPB8 could suppress aggregation of 4MBP by binding and stabilizing partially folded states populated by MBP monomers during force relaxation, thereby shielding them from interactions with other MBP monomers that produce misfolded or aggregated structures.^58^ If this was the case, one should observe an increased frequency of small intermediate folded states and a concomitant decreased frequency of native state,^58^ but this is not what we observe (Figures S3 and S4). In fact, the fraction of amino acids that end up in a small structure (Figure 1G) and the fraction of stretching traces characterized by the presence of small jumps (Figure 2E) do not increase significantly in the presence of HSPB8 WT or K141E. A related scenario in which HSPB8 binds to near-native structures, as shown for HSP70 and HSP42,^31,34^ is inconsistent with the fact that HSPB8 WT and K141E both do not alter the mechanical stability of the core structures (Figure 3, left column) or the frequency of visited states in sMBP (Figures S3 and S4). These data challenge the application to HSPB8 of model mechanisms proposed for the anti-aggregation activity of other chaperones in previous investigations.

Our observations, in particular the lack of effect on core refolding frequencies (in sMBP and 4MBP) while aggregation is suppressed (4MBP), rather suggest a model in which HSPB8 interacts with early off-pathway aggregated species and hence limits their development into larger and more stable non-native structures, while avoiding interactions with on-pathway folded states. Consistent with this hypothesis, the size of the weak aggregates is indeed smaller in the presence of HSPB8 WT (Figure S2) and their unfolding forces are lower (Figure 3, right column). Moreover, the interaction between HSPB8 and non-native structures at the onset of aggregation may also produce loose structures that are mechanically weak. These weak structures may not yield distinct unfolding steps in our data upon application of mechanical force, and hence may explain the sharp increase in the number of completely unstructured cycles in the presence of HSPB8 WT, Figure 1G. A preferential interaction of HSPB8 with misfolded states is actually not so surprising if we consider that the structural and hydrophobicity features of the misfolded states may differ significantly from those of the molecular states visited by the substrate protein during native folding. One might speculate that misfolding gives rise to compact, relatively stable, and highly hydrophobic local structures that exhibit enhanced affinity towards HSPB8. Remarkably, a quite similar mechanism of action has been proposed for the sHSP α-crystallin/HSPB4. Results from NMR and light spectroscopy experiments suggest that α-crystallin/HSPB4 does not recognize native folding intermediates of its substrate protein. Rather, it interacts with misfolded states that are on the path to aggregation.^69−72^ The remarkable similarities between the behaviors of HSPB8 and α-crystallin/HSPB4 might reveal an evolutionary selected mechanism of action common to other sHSPs.

Our data on the K141E mutant are also consistent with HSPB8 interacting specifically with protein aggregates and not protein monomers, as this mutation specifically impacts aggregate formation without impacting monomer behavior (Figures 1G and 2E). In contrast, if the function of HSPB8 would involve balancing a trade-off between aggregation suppression and native folding, as is the case for common existing models,^57,58,73^ one would expect mutations that affect aggregation between proteins to also impact the folding of isolated monomers. As the mutant K141E displays the same behavior of HSPB8 WT in the aggregation-free context provided by the monomer assays, not affecting either the unfolded or the folded states of sMBP, it is reasonable to think that the missense mutation in the α-crystallin domain of HSPB8 hampers its ability to recognize and interact with emerging misfolded/aggregated species. The pathogenic implications of K141E have been attributed to the mutation hindering the HSPB8-BAG3 interactions, as well as inhibiting HSPB8 dimerization.^42,74^ HSPB8 monomers present unstructured domains at their N- and C-termini, which have been suggested to be responsible for binding non-native conformers, and indeed may do so in a versatile and labile, low-affinity manner.^50^ One may speculate, among other effects, that WT HSPB8 dimers can interact with early MBP aggregates with sufficient affinity due to the cooperative binding of the additional unstructured domains, which is known to have a more than additive effect on affinity. Overall, our results indicate that the mutation also decreases its affinity for non-native species, which in turn reduces, but does not eliminate, its ability to hinder the growth of large aggregated structures.

## Conclusions

Our data paint a specific picture of HSPB8 function, in which it interferes at the onset of aggregation and interacts in a labile manner to deter further growth, without affecting native folding. Interaction with the HSC70/HSP70 co-chaperone BAG3 would then favor the autophagy-mediated degradation of the HSPB8-bound early-stage aggregates. Thus, HSPB8 is emerging as a member of the family with higher affinity for “mini-aggregates”. This interpretation is supported by previous findings showing that it fails to prevent the aggregation of long expanded polyglutamine proteins, which rapidly form large aggregates, while effectively suppressing aggregation of small expanded polyglutamine proteins, whose aggregation rate is slower.^75^ Whether in the cellular context HSPB8 binds with higher affinity to intermediate oligomeric species formed by these aggregation-prone proteins, which are considered to represent the more toxic species, remains to be determined.

## Materials and Methods

### Protein Expression and Purification

HSPB8 and HSPB8-K141E were subcloned in a pET11d vector as N-terminal 3C protease-cleavable GST fusion proteins. HSPB8 proteins were expressed and purified from BL21AI *E. coli* (Invitrogen). Expression was induced by adding 0.15 mM IPTG and 0.2% Arabinose for 4 h at 30 °C. Bacteria were lysed in 1× PBS, 1 mM DTT supplemented with Protease Inhibitor Cocktail (Calbiochem), PMSF, and Benzonase. The lysate was GST purified with Protino GST column (Machery-Nagel). Eluates were dialyzed with a 10 kDa MWCO membrane against 1× PBS, 1 mM DTT, and cleaved with PreScission protease. Reverse GST purification was used to remove cleaved-off GST. HSPB8 proteins were subjected to ResourceQ ion-exchange chromatography, concentrated using Amicon Ultra centrifugal filters (Merk Millipore), and dialyzed to HSPB8 buffer (20 mM Hepes pH 7.4, 20 mM KCl, and 1 mM DTT). Aliquots were flash-frozen and stored at −80 °C. To prepare HSPB8 for fluorescent labeling, concentrated aliquots were reduced with 100 mM DTT and purified by reversed-phase high-performance liquid chromatography (RP-HPLC) using a ZORBAX 300SB-C3 column (Agilent), followed by lyophilization. Lyophilized HSPB8 was resuspended in labeling buffer (0.1 M potassium phosphate, 1 M urea, pH 7.0) and labeled overnight at 4 °C using Cy3B maleimide (donor) (Cytiva) (0.7:1 dye-to-protein ratio). The reaction was quenched using DTT, and RP-HPLC was then used to remove unreacted dye and unlabeled and double donor-labeled constructs. Single-labeled protein was lyophilized overnight, then resuspended in labeling buffer, and labeled overnight at 4 °C using excess CF660R maleimide (acceptor) (Sigma), and the reaction was quenched and purified as before.

### Protein–DNA Constructs

N- and C-terminal MBP cysteines were coupled with maleimide single-strand DNA oligos of 20 bp in length, for 1 h at 37 °C. Double-stranded DNA strands of lengths of 2.5 and 1.3 kbp were generated by PCR from a pUC19 plasmid (NEB), using either a double digoxigenin- or a biotin-labeled primer on one end, and a phosphoprimer on the other end, which were subsequently purified using a QIAquick PCR purification kit (Qiagen). Next, we digested the phosphorylated strand using Lambda exonuclease (NEB), for 2 h at 37 °C. Subsequent purification was performed using an Amicon 30 kDa MWCO filter (Merck). Using Deep Vent exo-DNA polymerase (NEB) and a 20 nt primer, which was positioned more upstream than the phosphoprimer from the PCR, we filled up the second DNA strand, while yielding a 20 nt overhang at one of the ends, which complements the 20 nt oligo, previously coupled to the two MBP termini. The resulting DNA strands (one containing a double digoxigenin at one end, and one containing a biotin at one end) were mixed with the MBP-oligo chimera as well as with T4 ligase (NEB) and incubated for 30 min at 16 °C followed by 30 min on ice. The resulting DNA-MBP-DNA hybrid was flash-frozen and stored at −80 °C until measurement.

### Optical Tweezers Experiments

All assays were performed using a custom-built optical tweezers instrument with a dual-beam laser trap of 840 nm wavelength.^64,76^ The two substrates, 4MBP and sMBP, flanked by 1333 bp DNA handles were sandwiched between an antidigoxigenin-coated bead (3.10 μm) which was caught in the optical trap and a streptavidin-coated bead (2.18 μm), which was held by a micropipette. All experiments were carried out at ambient temperature in 50 mM HEPES, 100 mM KCl, 5 mM MgCl_2_, 0.05% sodium azide, pH 7.5 in the absence or presence of 5 μM HSPB8 or 5 μM HSPB8-K141E. At the beginning of the experiment, the two beads are bought in contact deliberately and slowly to facilitate tether formation. The substrates are then put through multiple cycles of mechanical denaturation and relaxation by moving the micropipette away from and toward the optical trap, respectively.^77^ The pulling and relaxation cycles were punctuated by a 5 s waiting period after relaxation at 0 pN to allow refolding/aggregation to take place. HSPB8 and HSPB8-K141E were both diluted in the same buffer and introduced in the fluid chamber by means of a fluid pump.

### Data analysis

The most informative feature of the force vs extension stretching curves are the ″jumps″: sudden drops in force accompanied by an increase of the extension, which signal the breaking of some molecular structure. In order to gather insight into the nature of such events, it is necessary to estimate the size of the broken structure in terms of the number of amino acids involved. While the elastic properties of a chain of denatured residues are very well described by a worm-like chain (WLC), it is however notoriously harder to fit the same model (actually, the extensible version of it) to the behavior of the handles of the molecular construct. We have devised a novel solution to this well-known problem that consists in combining the experimental traces of stretching and relaxation to build an empirical reference curve that does not rely on a WLC fitting for the handles. The result of this construction are the gray lines shown in Figures 1C–G and 2B–D. The interested reader will find a detailed description of this method in ref (78).

### Single-Molecule Spectroscopy

All single-molecule fluorescence experiments were conducted at 23 °C using a MicroTime 200 (PicoQuant) connected to an Olympus IX73 inverted microscope. The donor dye was excited with a 520 nm diode laser at 40 μW, using pulsed interleaved excitation (PIE) with a 640 nm diode laser at 20 μW. Excitation and emission light was focused and collected using a 60x water objective (UPLSAPO60XW, Olympus). Emitted fluorescence was focused through a 100 μm pinhole before being separated first by polarization and then by emission wavelengths into four single-photon avalanche diodes. The arrival time of detected photons was recorded with a MultiHarp 150P time-correlated single photon counting (TCSPC) module (PicoQuant). Experiments were performed in μ-Slide sample chambers (Ibidi) in the same buffer as the optical tweezers experiments with added 143 mM 2-mercaptoethanol (Sigma) for photoprotection and 0.01% (v/v) Tween-20 (AppliChem) to reduce surface adhesion. Data for transfer efficiency histograms were collected from 50–100 pM freely diffusing double-labeled HSPB8. All data were analyzed using the Mathematica scripting package “Fretica” (https://schuler.bioc.uzh.ch/programs/). Fluorescence bursts were first identified by combining all detected photons with less than 100 μs interphoton times. FRET efficiencies within each fluorescence burst were calculated according to *E* = *n*′*A*/(*n*′*A* + *n*′*D*), where *n*′*A* and *n*′*D* are the number of acceptor and donor photons, respectively. The number of photons were corrected for background, direct acceptor excitation, channel crosstalk, differences in dye quantum yields, and photon detection efficiencies.^79^ To extract mean FRET efficiencies, histograms of all FRET efficiencies were fitted to an appropriate number of Gaussian or logNormal distribution function. To determine the diffusion time of labeled HSPB8, we performed fluorescence correlation spectroscopy^80^ in the absence and presence of MBP by correlating the fluctuations of donor and acceptor fluorescence intensity in an smFRET experiment using photon lag times τ from 10^–6^ to 1 s.

## Supporting Information Available

Single-molecule spectroscopy data, graphs of distributions of aggregate size, and visited molecular states (PDF) Detailed description of the statistical significance of the data in Figures 1G and 2E (PDF)

## Footnotes

a According to one-way ANOVA test at a 5% significance level. For details about the statistical treatment of the data in Figure 1G, see Section 1 in the Supporting Information.
