# Supplementary material for: Human Small Heat Shock Protein B8 Inhibits Protein Aggregation without Affecting the Native Folding Process
Source: J Am Chem Soc. 2023 Jul 6;145(28):15188–96. doi: 10.1021/jacs.3c02022 (PMC10360156; doi:10.1021/jacs.3c02022)
Supplement: Supplementary file 1 — ja3c02022_si_001.pdf [file ja3c02022_si_001.pdf]

Supporting Information

The human small heat shock protein B8 inhibits protein aggregation without affecting the native folding process

Dhawal Choudhary[1,2,4], Laura Mediani[3], Mario J. Avellaneda[4], Sveinn Bjarnason[5], Simon Alberti[6], Edgar E. Boczek[6], Pétur O. Heidarsson[5], Alessandro Mossa[2,7*], Serena Carra[3*], Sander J. Tans[4*], Ciro Cecconi[1,2*]

1 Department of Physics, Informatics and Mathematics, University of Modena and Reggio Emilia, 41125 Modena, Italy;

2 Center S3, CNR Institute Nanoscience, Via Campi 213/A, 41125 Modena, Italy;

3 Department of Biomedical, Metabolic and Neural Sciences, and Centre for Neuroscience and Neurotechnology, University of Modena and Reggio Emilia, via G. Campi 287, 41125 Modena, Italy,

4 FOM institute AMOLF, Science Park 104, 1098 XG Amsterdam, The Netherlands,

5 Department of Biochemistry, Science Institute, University of Iceland, Sturlugata 7, 102 Reykjavík.

6 Max Planck Institute of Molecular Cell Biology and Genetics, Pfotenhauerstr. 108, D-01307 Dresden.

7 INFN Firenze, via Sansone 1, 50019 Sesto Fiorentino, Italy

Significance of the experimental data

In Fig. 1G we measure the ratio of residues that end up in one of the five states **U**nstructured, **S**mall, **C**ore, **W**eak aggregate, and **T**ight aggregate in our experiments with 4MBP substrate. The statistical treatment of this data requires some care. We assume that results obtained for individual molecules are statistically independent, while the same cannot be assumed about single traces within the same dataset, due to the presence of aggregate structures that can survive from one trace to the next. However, it seems reasonable to weight the results obtained from individual molecules with the number of traces in that dataset. Table S1 shows the 18 datasets used for this study.

For each one of our five observables (U, S, C, W, T frequencies) we apply a one-way ANOVA test (the standard choice when comparing more than two groups of data) with the null hypothesis that the frequency is the same under all three experimental conditions. The "between-group" degree of freedom is 2, while the "within-group" degree of freedom is 15, so the critical value of the F-ratio at the 5% significance level is 3.68. Table S2 summarizes the test results for the 5 observables. As stated in the main text of the article, the frequency of forming Core and Small structures is essentially the same, while the presence of the chaperone (either in its wild-type or mutant form) significantly affects the formation of aggregates.

The statistical treatment of the classification of traces for the monomer data (Fig. 2E) is somewhat different because in this case aggregation is not possible, and we can regard the single traces as statistically independent. However, nothing prevents us from repeating the same one-way ANOVA test, again with the null hypothesis that the frequency of the various kind of traces is the same under all three experimental conditions, Table S3. In this case the "between-group" degree of freedom is 2, while the "within-group" degree of freedom is 16, so the critical value of the F-ratio at the 5% significance level is 3.63. Table S4 shows the results of the test: there is no statistical evidence in support of rejecting the null hypothesis for any kind of traces.

	U	S	C	W	T	weight
**no, 1**	0.196180	0.169827	0.0883970	0.502559	0.0430373	14
**no, 2**	0.192968	0.158974	0.0878523	0.373971	0.186235	61
**no, 3**	0.245725	0.187841	0.0673524	0.391123	0.107958	26
**WT, 1**	0.606233	0.166888	0.0626452	0.164234	0	32
**WT, 2**	0.503401	0.232768	0.0953260	0.139412	0.0290934	32
**WT, 3**	0.439527	0.160606	0.140124	0.259743	0	30
**WT, 4**	0.530701	0.160451	0.0662906	0.201062	0.0414952	30
**mut, 1**	0.328633	0.205541	0.0571914	0.368281	0.0403534	16
**mut, 2**	0.245205	0.137454	0.0609536	0.529055	0.0273328	21
**mut, 3**	0.352473	0.203872	0.123112	0.300675	0.0198681	17
**mut, 4**	0.448131	0.368650	0.0611222	0.116125	0.00597213	9
**mut, 5**	0.455236	0.250936	0.108772	0.185056	0	25
**mut, 6**	0.269741	0.170537	0.0587259	0.382216	0.118779	6
**mut, 7**	0.199679	0.226499	0.0922915	0.481531	0	12
**mut, 8**	0.247180	0.195421	0.0601637	0.497235	0	6
**mut, 9**	0.360012	0.214078	0.154203	0.271706	0	13
**mut, 10**	0.432497	0.217530	0.130965	0.219008	0	22
**mut, 11**	0.319588	0.246184	0.154233	0.246216	0.0337789	21

Table S1. Datasets used for Fig. 1G. Each row refers to one dataset, comprised of experimental traces measured on one individual 4MBP molecule. The statistical weight in the right-most column is the number of traces in the dataset. In the first column, the experimental conditions are labeled as “no” for experiments in the absence of chaperone, “WT” for experiments in the presence of wild-type HSPB8, “mut” for experiments in the presence of K141E mutant of HSPB8.

	***F*-ratio**	***p* value**	**outcome**
**Unstructured**	17.245	0.00012936	H0 rejected
**Small**	2.94417	0.0834459	H0 not rejected

**Core**	0.69868	0.512719	H0 not rejected
**Weak aggregate**	4.02132	0.0399663	H0 rejected
**Tight aggregate**	16.3384	0.000171151	H0 rejected

Table S2. Results of the one-way ANOVA test applied to the data of Table S1. The null hypothesis is that the mean frequency of each state is the same across all datasets.

	**U**	**S**	**C**	**N**	**L**	**total**
**no, 1**	3	6	7	3	1	20
**no, 2**	4	6	8	5	0	23
**no, 3**	7	4	8	4	1	24
**no, 4**	6	12	8	8	1	35
**no, 5**	0	1	3	2	1	7
**no, 6**	7	4	6	6	2	25
**no, 7**	4	1	11	3	1	20
**no, 8**	5	0	4	4	0	13
**WT, 1**	7	7	2	5	1	22
**WT, 2**	1	3	8	8	1	21
**WT, 3**	6	9	5	7	1	28
**WT, 4**	8	10	22	7	1	48
**WT, 5**	18	10	12	3	0	43
**WT, 6**	8	5	21	7	1	42
**mut, 1**	6	6	13	7	1	33
**mut, 2**	4	3	10	3	0	20
**mut, 3**	8	2	7	5	6	28
**mut, 4**	4	8	5	8	0	25
**mut, 5**	8	7	8	11	2	36

Table S3. Datasets used for Fig. 2E. Each row refers to one dataset, comprised of experimental traces measured on one individual sMBP molecule. In the first column, the experimental conditions are

labeled as “no” for experiments in the absence of chaperone, “WT” for experiments in the presence of wild-type HSPB8, “mut” for experiments in the presence of K141E mutant of HSPB8.

	***F*-ratio**	***p* value**	**outcome**
**Unstructred**	0.104659	0.901243	H0 not rejected
**Small**	0.474157	0.630882	H0 not rejected
**Core**	0.133597	0.875908	H0 not rejected
**Native**	0.806600	0.463718	H0 not rejected
**Large**	0.817112	0.459313	H0 not rejected

Table S4. Results of the one-way ANOVA test applied to the data of Table S3. The null hypothesis is that the mean frequency of each kind of trace is the same across all datasets.

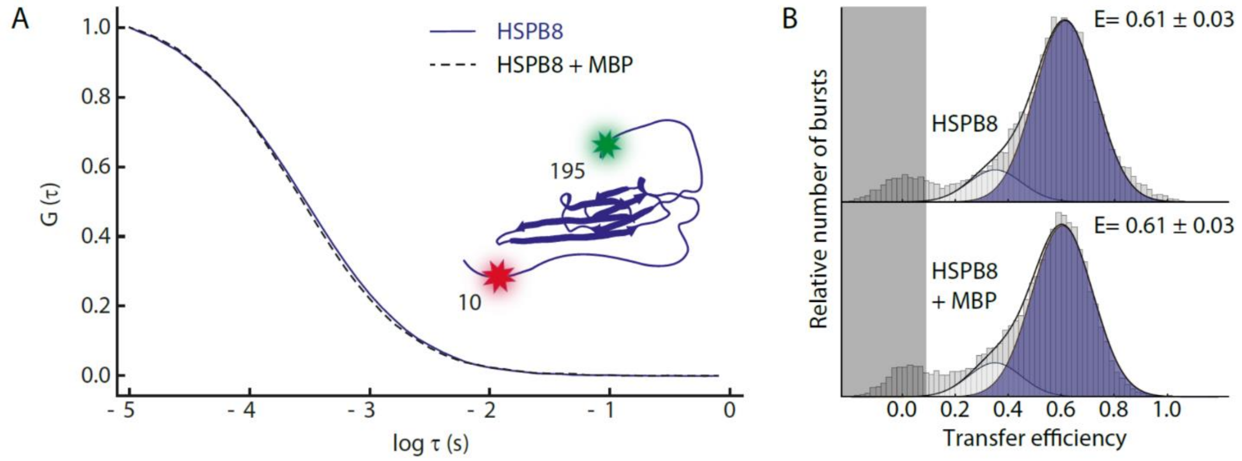

Figure S1. Single-molecule spectroscopy analysis of HSPB8. A) Normalized donor-acceptor cross-correlation of fluorescently labelled HSPB8 labelled at residues 10 and 195. Diffusion time through the confocal volume remains the same for HSPB8 (50-100 pM) in the absence and presence of sMBP (5 μM). B) Single-molecule transfer efficiency histograms of HSPB8 labelled at the same residues as in panel A, in the absence (number of molecules = 11872) and presence of 5 μM sMBP (number of molecules = 19158). No change in mean transfer efficiency (E) can be observed upon the addition of sMBP. The grey shaded box indicates a residual donor-only population originating from molecules lacking an active acceptor.

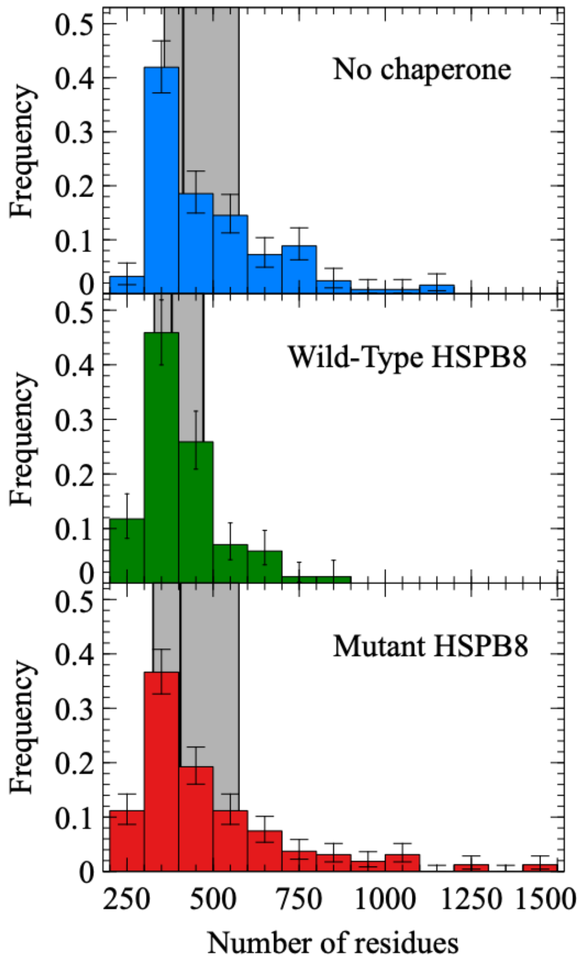

Figure S2. Distribution of the estimated size of weak aggregates in three distinct experimental settings: 4MBP in the absence of chaperone (top row), in the presence of the wild-type HSPB8 (middle row), or in the presence of the K141E mutant HSPB8 (bottom row). As a guide to the eye, the region between the first and third quartile of the distribution has been highlighted by a grey rectangle. Also, the position of the median is represented by a vertical line. The presence of the wild-type chaperone significantly alters the distribution by favoring shorter aggregates. The mutant form increases the probability of very short (about 250 residues) aggregates in a similar way to the wild-type, but is not able to suppress the long tail of large aggregates. Histograms are based on 107 traces (3 individual molecules) in the dataset without chaperone, 132 traces (4 molecules) in the dataset with wild-type chaperone, and 190 traces (11 molecules) in the dataset with mutant chaperone.

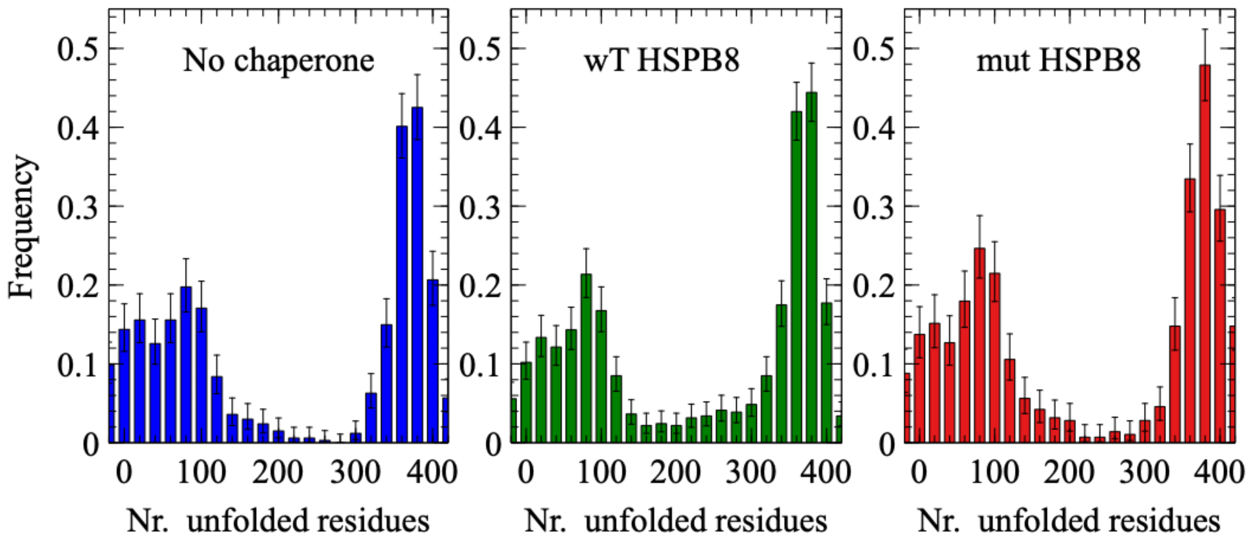

Figure S3. Frequency of visited states in Maltose Binding Protein (MBP) monomer experiments. For each stretching trace and for each experimental point, the number of unfolded residues is estimated by comparison with the grid of reference curves built upon the baseline. The points with force below 2 pN are discarded because in that regime the reconstruction is too noisy. All other points get binned on a grid: a "visit" is recorded in any single trace if the tally in the corresponding bin is at least 10 points. Finally, the histogram of visited states is normalized dividing by the number of traces in each dataset: 179 traces (8 individual molecules) for the dataset without chaperone, 214 traces (6 molecules) for the dataset with wild-type chaperone, and 155 traces (5 molecules) for the dataset with the mutant chaperone.

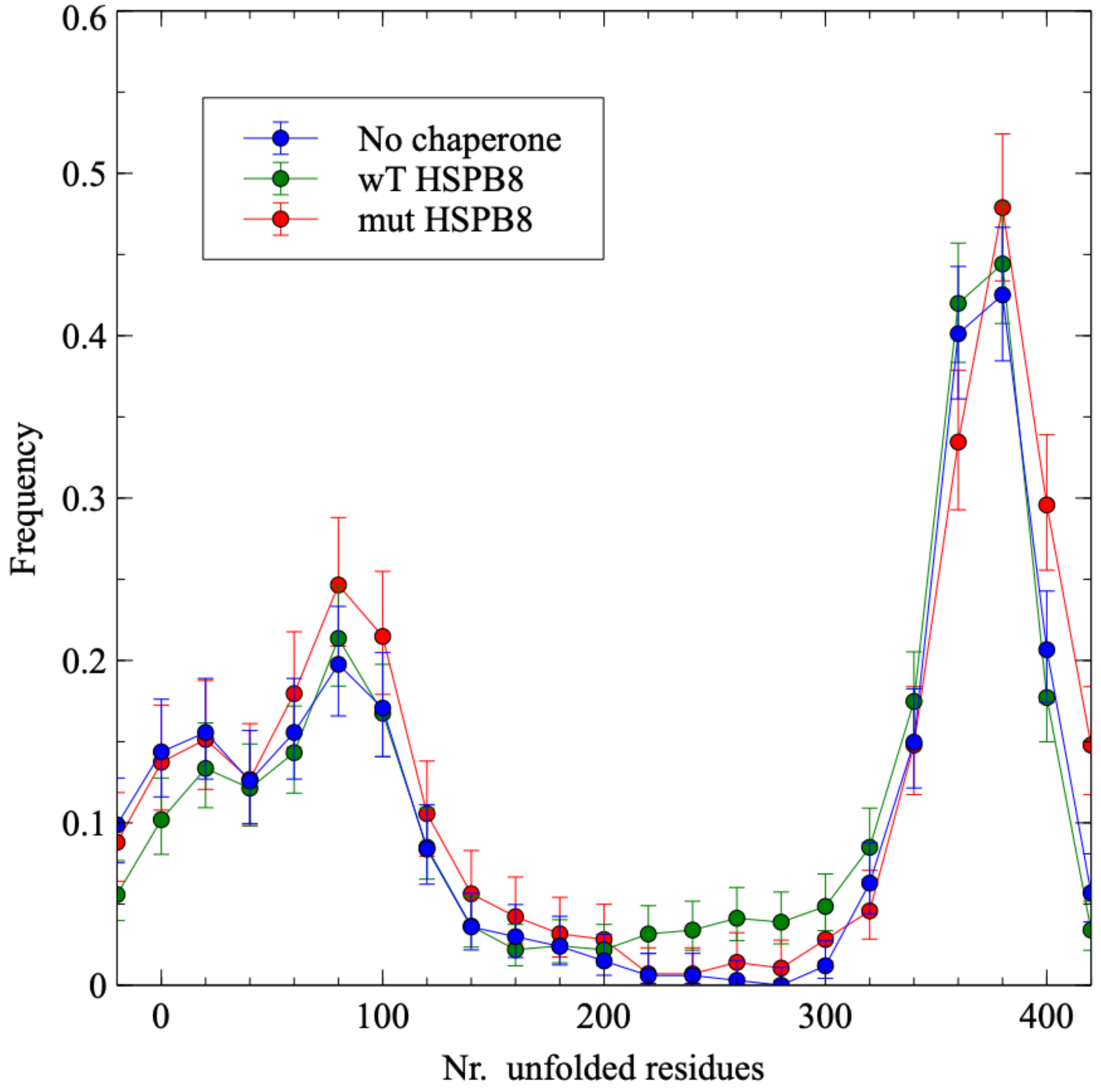

Figure S4. The same information as Figure S3, but the histograms from the three datasets are represented in the same graph for ease of comparison among them. The error bars signal the 68.3% central confidence interval.
